# Paracoccidioidomycosis: a benign cause of avid-FDG-PET/CT bone lesions

**DOI:** 10.1016/j.bjid.2024.103871

**Published:** 2024-09-24

**Authors:** Vitor Cassiano Albuquerque Maiolo, Paulo Henrique Rosado de Castro, Edson Marchiori, Miriam Menna Barreto, Rosana Souza Rodrigues

**Affiliations:** a Universidade Federal do Rio de Janeiro (UFRJ), Departamento de Radiologia, Rio de Janeiro, RJ, Brazil; b Universidade Federal do Rio de Janeiro, Departamento de Medicina Nuclear, Rio de Janeiro, RJ, Brazil; c Instituto D'Or de Pesquisa e Ensino, Rio de Janeiro, RJ, Brazil

An 18-year-old man came to the emergency room due to fever, productive cough, asthenia, 30 kg weight loss, generalized lymphadenopathy, and migratory polyarthritis in the last 6-months. Laboratory test findings were unremarkable. A blood test for human immunodeficiency virus was negative. Computed Tomography (CT) showed multiple well-delimited osteolytic lesions with no sclerotic halo or contrast enhancement affecting the sternum, elbows, ilium, sacroiliac joint, knees, and ankles, with no evidence of periosteal reaction (Fig. 1). There was no sign of pulmonary involvement. Metastatic disease was suspected. On Positron Emission Tomography (PET)/CT, Fluorodeoxyglucose (FDG) uptake was intense in the osteolytic lesions (maximum standard uptake = 14.3; Fig. 2) and some mediastinal lymph nodes. The diagnosis of Paracoccidioidomycosis (PCM) was confirmed through direct mycological examination of a lymph-node biopsy sample, which demonstrated spherical, thick-walled yeast cells of variable size, with peripheral buds protruding from a central cell, a pattern called the “pilot's wheel”, characteristic of *Paracoccidioides brasiliensis. Paracoccidioides spp*. also grew in the culture after 4-weeks. The patient was treated with antifungal drugs and the lesions improved.

**Fig. 1:**
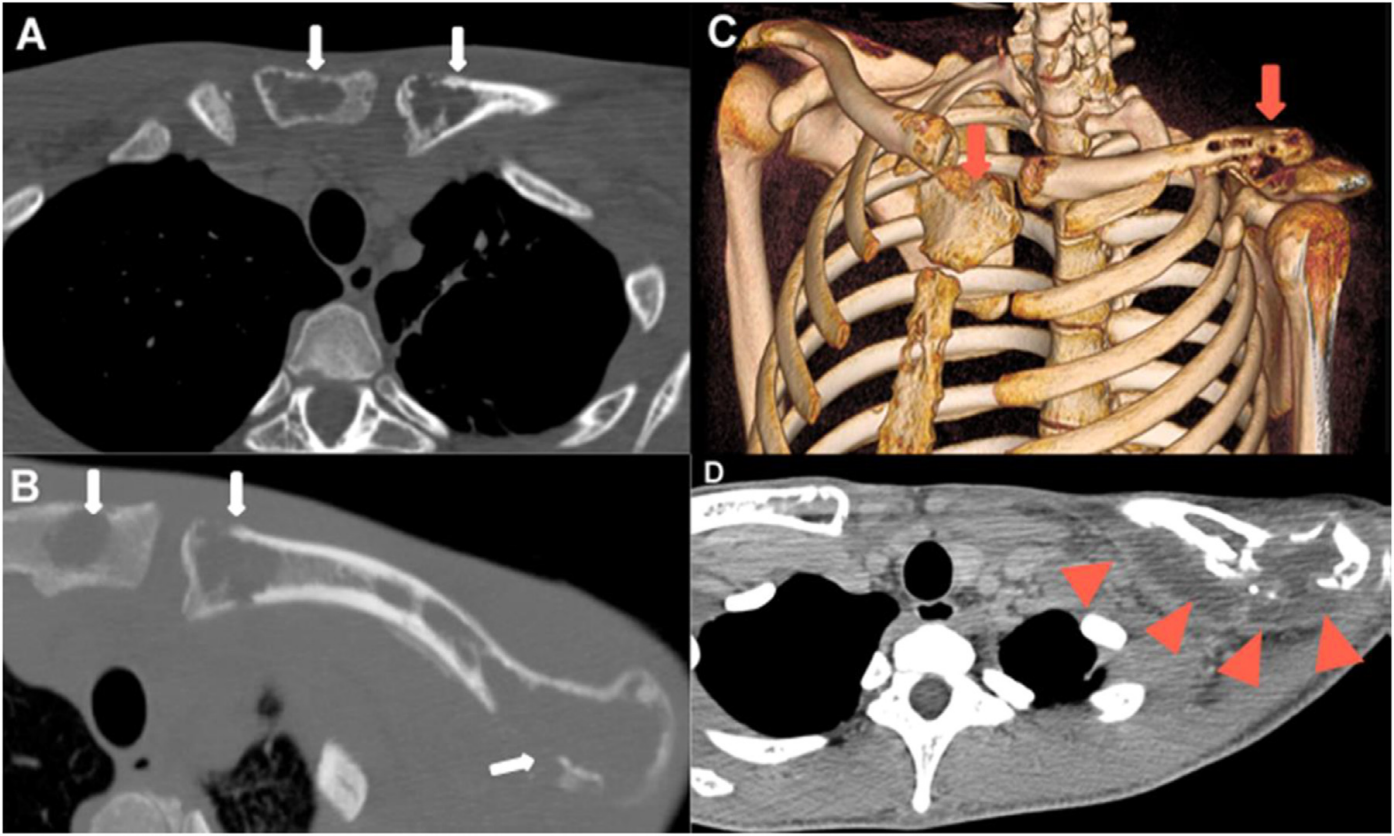
(A‒B) CT images obtained in the axial plane and bone window showing lytic lesion signs with cortical destruction of the left clavicle and manubrium and no periosteal reaction (white arrows). (C) Volume-rendered 3D image also demonstrating multiple osteolytic lesions with cortical rupture of the manubrium, sternum, and left clavicle (red arrows). (D) CT image obtained in the axial plane and soft-tissue window depicting fluid collection around the bony destruction of the left clavicle (red arrowheads).

**Fig. 2:**
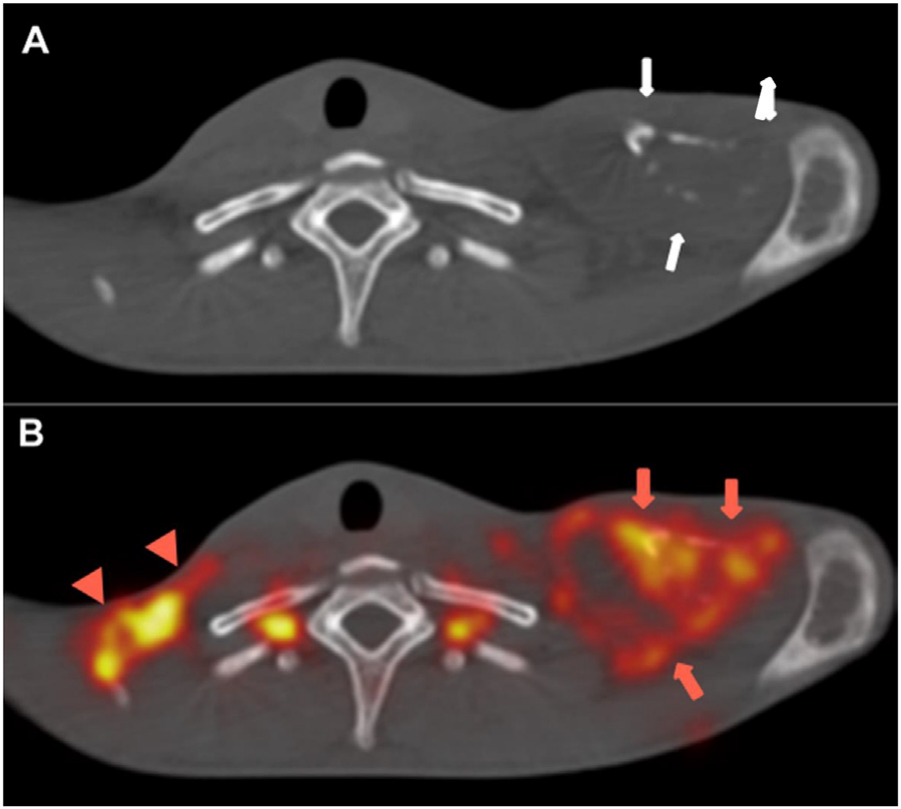
(A) Axial CT image (bone window) showing large erosions of the left clavicle with soft tissue swelling (white arrows). (B) Axial fused FDG PET/CT image showing FDG uptake (red arrows) of the lytic lesions in the left clavicle and in the fluid collection around the bone (SUVmax = 14.3). There is also intense uptake in the right supraclavicular lymph nodes (arrowheads).

PCM is classified into two main forms based on its clinical aspects.^1^, ^2^, ^3^ The acute or subacute (juvenile) form most frequently affects children, teenagers, and young adults. The chronic (adult) form is the most common and affects predominantly individuals aged >30-years. Thus, the present case can be classified as acute/subacute. PCM can affect any bone, but it most frequently affects the clavicle, ribs, scapula, and sternum. The most common radiographic characteristics are lytic lesions with no marginal sclerosis and little or no periosteal reaction.^1^, ^2^, ^3^, ^4^

In conclusion, lytic bone lesions that are FDG avid on PET/CT are not necessarily malignant; there may be overlap with infectious diseases, which often poses a diagnostic challenge for clinicians and radiologists.

## Conflicts of interest

The authors declare no conflicts of interest.
